# Healthy Ageing Through Yoga: Bridging Prevention, Function, and Rehabilitation

**DOI:** 10.7759/cureus.111205

**Published:** 2026-06-20

**Authors:** Raktim Swarnakar

**Affiliations:** 1 Physical Medicine and Rehabilitation, All India Institute of Medical Sciences, Deoghar, Deoghar, IND

**Keywords:** functional status, healthy ageing, prevention, rehabilitation care, yoga benefits

## Abstract

Population ageing is a growing global challenge that requires strategies aimed not only at increasing lifespan but also at preserving health, independence, and quality of life. Healthy ageing emphasizes the maintenance of physical, mental, and social well-being, with particular focus on sustaining functional ability throughout later life. Yoga, an ancient mind-body practice integrating physical postures, breathing techniques, and meditation, has gained increasing recognition as a potential contributor to these goals. This editorial explores the role of yoga in promoting healthy ageing through its contributions to prevention, functional maintenance, and rehabilitation. Yoga may encourage physical activity, improve mobility and balance, support psychological well-being, and facilitate participation in daily life. In addition, it may help preserve functional independence and complement rehabilitation efforts in individuals with age-related health conditions and disabilities. Its adaptability allows practices to be modified according to individual needs and functional abilities, making it applicable across diverse ageing populations. By spanning the continuum from health promotion to rehabilitation, yoga offers a practical approach that aligns with contemporary function-centered and person-centered models of care. While evidence supporting yoga for healthy ageing continues to grow, findings remain heterogeneous, and implementation requires appropriate adaptation, safety screening, and guidance from trained instructors, particularly for older adults with functional limitations or multiple comorbidities. Recognizing yoga as more than a wellness practice may contribute to a more comprehensive approach to ageing well and support broader healthy ageing goals.

## Editorial

Population ageing is one of the defining demographic trends of the 21st century. Advances in healthcare have increased life expectancy worldwide, but longer lives do not necessarily translate into healthier lives. Healthy ageing requires maintaining physical, mental, and social well-being while preserving the ability to function independently and participate in meaningful activities. As the world prepares to observe the International Day of Yoga on June 21, 2026, under the theme “Yoga for Healthy Ageing,” attention is being drawn to the potential of yoga to support healthy ageing through prevention, functional maintenance, and rehabilitation [1]. This theme underscores the potential of yoga as a practical and accessible approach to support health and well-being throughout the ageing process.

Healthy ageing extends beyond the prevention of disease. It encompasses maintaining mobility, independence, participation, and quality of life despite age-related physiological changes. Older adults frequently experience declines in muscle strength, flexibility, balance, endurance, and coordination, all of which can contribute to reduced functional capacity and increased risk of disability. Consequently, there is a need for interventions that address these challenges while remaining safe, affordable, and adaptable to diverse populations.

Yoga, an ancient mind-body practice that integrates physical postures (asanas), breathing techniques (pranayama), and meditation, has gained increasing attention as a health-promoting intervention. From a preventive perspective, yoga may help counteract several factors associated with unhealthy ageing. A systematic review and meta-analysis demonstrated that yoga-based exercise improves balance and mobility among individuals aged 60 years and older, highlighting its potential role in maintaining physical function and independence [2]. Improvements in flexibility, strength, and postural control may enable older adults to remain active and reduce the impact of age-related physical decline.

Beyond physical health, healthy ageing also requires attention to psychological and emotional well-being. Loneliness, anxiety, stress, and depressive symptoms are common among older adults and can negatively influence health outcomes and participation in daily activities. Yoga offers a holistic approach that combines physical activity with mindfulness and controlled breathing, thereby addressing both physical and mental dimensions of health. A systematic review of randomized controlled trials found that yoga-based exercise improved health-related quality of life and mental well-being in older adults [3]. These benefits may enhance self-confidence, resilience, and overall life satisfaction, which are essential components of healthy ageing.

Falls represent a major public health concern among older adults and are a leading cause of injury, hospitalization, and loss of independence. Effective strategies to improve balance and reduce fall risk are therefore critical. Emerging evidence suggests that yoga may contribute to fall prevention by enhancing balance, coordination, and physical performance. Furthermore, yoga has demonstrated favorable effects on bone health and factors associated with musculoskeletal resilience, reinforcing its potential role in promoting safe and active ageing [4]. Such findings support the inclusion of yoga within broader public health initiatives aimed at reducing disability and maintaining functional independence.

The benefits of yoga are not limited to prevention. The concept of healthy ageing places significant emphasis on preserving function, a central goal of rehabilitation medicine. Function encompasses an individual's ability to perform daily activities, engage in social roles, and participate fully in society. As healthcare systems increasingly shift toward person-centered care, interventions that promote function rather than simply treat disease are becoming increasingly important. Future efforts should focus on integrating yoga into community-based healthy ageing initiatives, rehabilitation services, fall-prevention and chronic disease management programs, while addressing barriers related to accessibility, instructor training, cultural adaptation, adherence, cost, and safety, particularly among frail older adults.

In this context, yoga serves as a bridge between health promotion and rehabilitation. Rehabilitation seeks to optimize functioning and quality of life in individuals experiencing impairments, activity limitations, or participation restrictions. Yoga may complement conventional rehabilitation approaches by addressing physical, psychological, and social dimensions of recovery. Adapted yoga programs have been explored in various chronic health conditions, including osteoarthritis, chronic musculoskeletal pain, stroke, Parkinson's disease, and cancer survivorship. The versatility of yoga allows practices to be tailored according to an individual's abilities and rehabilitation goals, making it accessible even for those with substantial functional limitations.

One of yoga's unique strengths is its ability to span the entire continuum of care [3,4]. An individual may begin practicing yoga as a preventive health measure during midlife, continue to use it to preserve mobility and function during older age, and later incorporate adapted yoga into rehabilitation following illness, injury, or surgery. Few interventions possess this capacity to support health across multiple stages of ageing and healthcare delivery.

Despite growing evidence, challenges remain regarding standardization, accessibility, and implementation. Further high-quality research is needed to determine optimal protocols, long-term outcomes, and strategies for integrating yoga into routine healthcare and rehabilitation services. Nevertheless, current evidence suggests that yoga is a promising intervention that aligns closely with the goals of healthy ageing. 

Although yoga offers the advantage of integrating physical, psychological, and mindful practices within a single intervention, it should be viewed as one of several approaches that may support healthy ageing across the continuum of prevention, functional maintenance, and rehabilitation. Its benefits are likely influenced by factors such as the style of yoga, frequency and intensity of practice, instructor expertise, and participant characteristics. Given the considerable heterogeneity among older adults, yoga programs should be individually adapted and implemented with appropriate screening, modifications, and professional supervision to ensure safety and effectiveness. Furthermore, yoga is best considered a complementary component of healthy ageing strategies rather than a stand-alone solution. Future research should focus on establishing optimal protocols, evaluating long-term adherence and safety, monitoring adverse events, and determining effectiveness in frail and medically complex older populations.

As populations continue to age globally, healthcare systems must prioritize interventions that preserve function, independence, and participation. Yoga represents a low-cost, culturally relevant, and increasingly evidence-based practice that can contribute to these objectives. By bridging prevention, function, and rehabilitation, yoga offers a holistic pathway toward healthy ageing and supports the vision of enabling older adults not only to live longer but also to live better.
